# Supplementary material for: Association of Pro-Inflammatory Diet, Smoking, and Alcohol Consumption with Bladder Cancer: Evidence from Case–Control and NHANES Studies from 1999 to 2020
Source: Nutrients. 2024 Jun 6;16(11):1793. doi: 10.3390/nu16111793 (PMC11174752; doi:10.3390/nu16111793)
Supplement: Supplementary file 1 [file nutrients-16-01793-s001.zip › nutrients-2991069-supplementary.pdf]

ADDITIONAL CHART SECTION

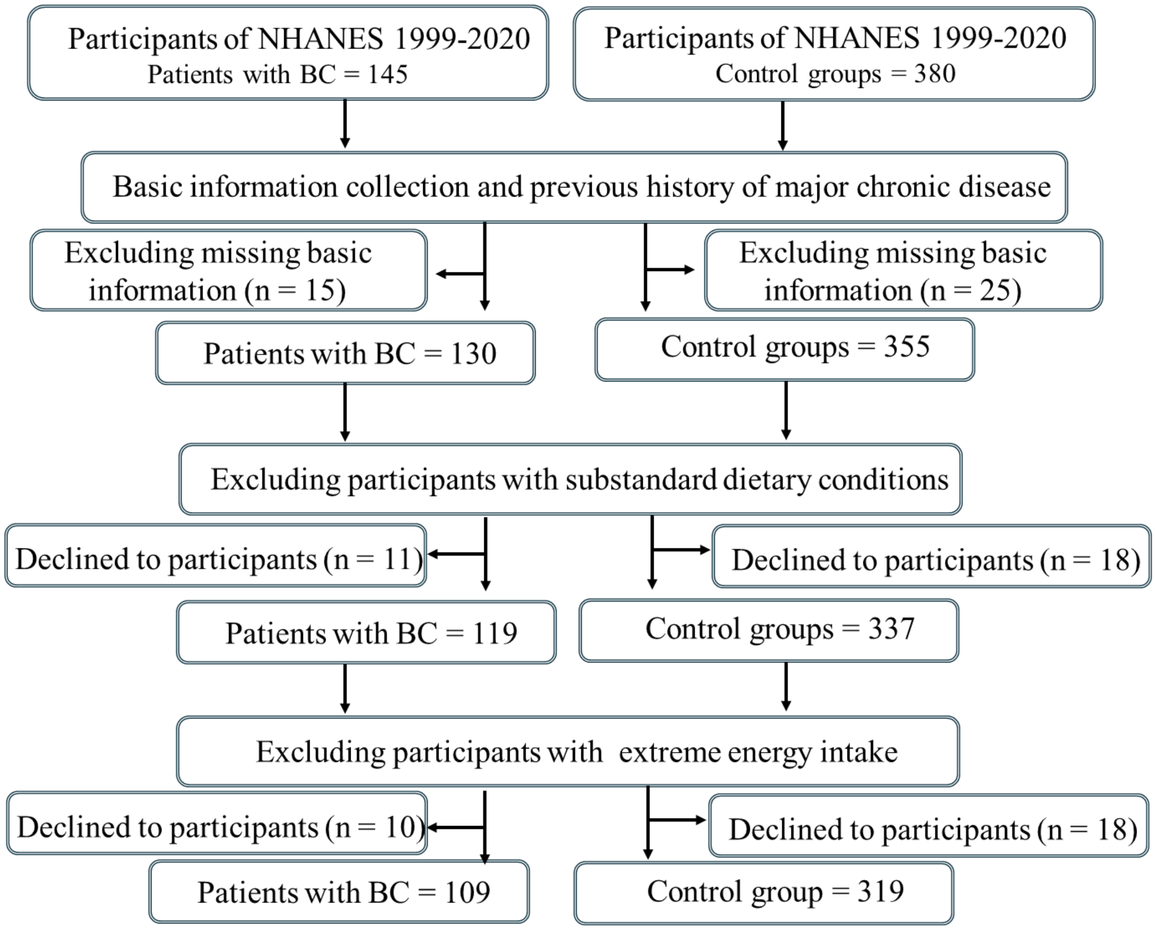

Supplementary Figure S1. Flow chart of the sample selection from NHANES 1999-2020.

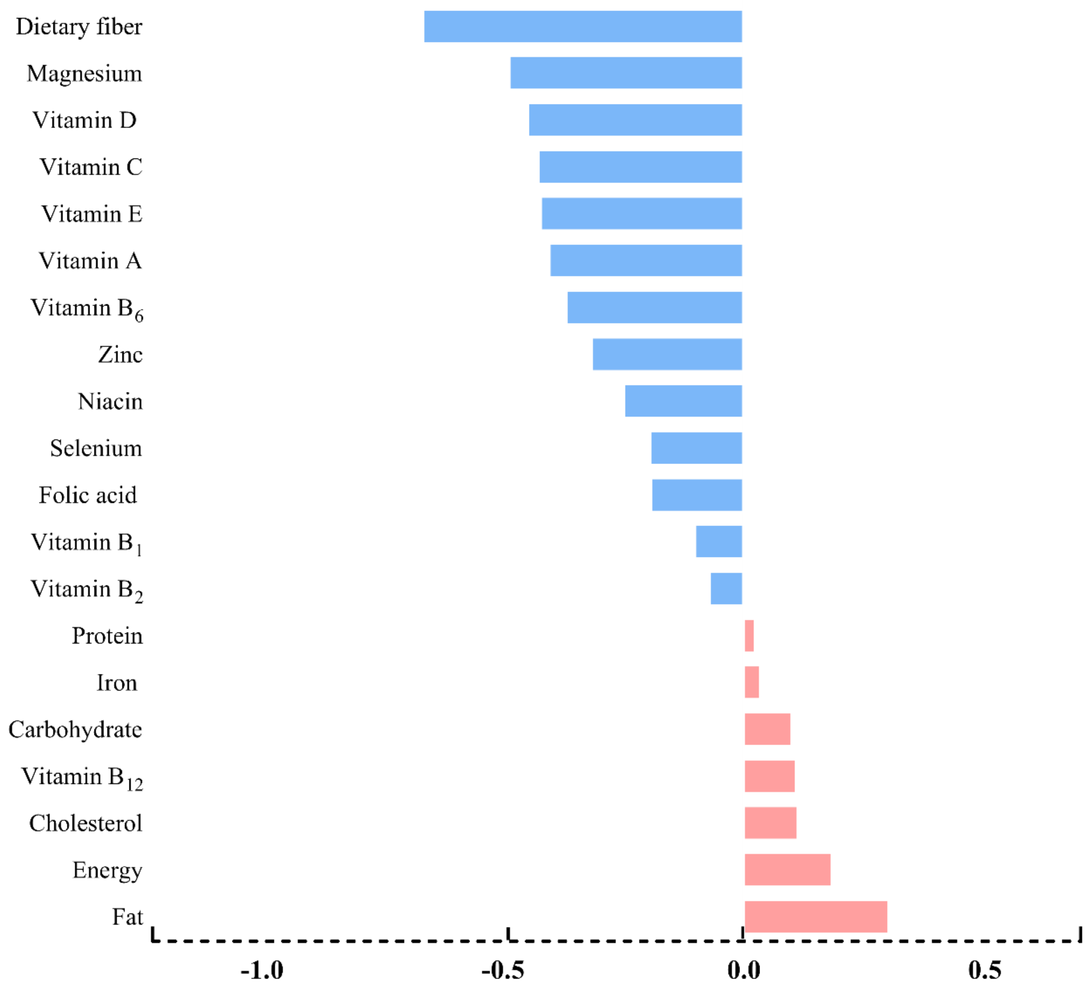

Supplementary Figure S2. Scoring of each food parameter to build the Dietary Inflammatory Index

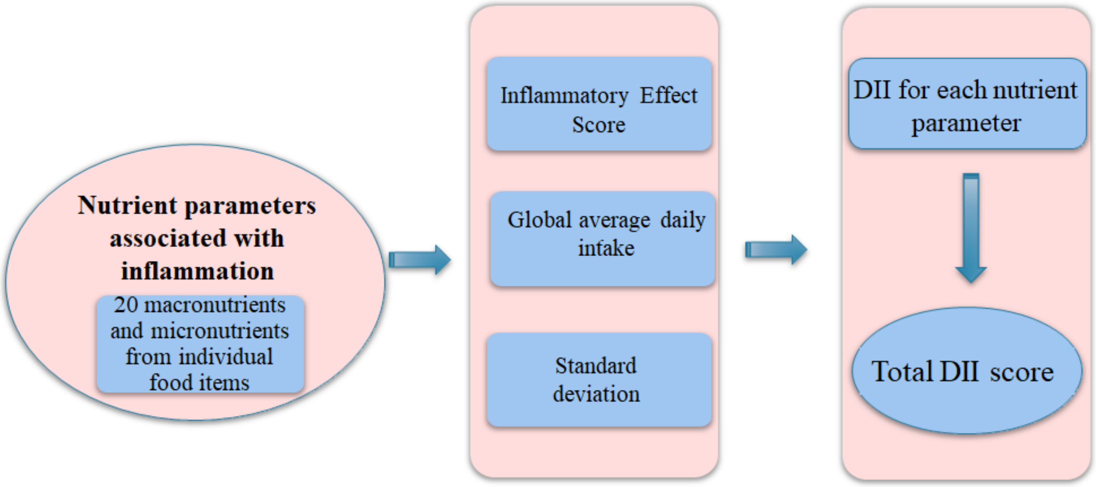

Supplementary Figure S3. Calculation of Dietary Inflammatory Index

Note: 20 food nutritional parameters: Energy (kcal), Carbohydrate (g), Protein (g), Fat (g), Dietary fiber (g), Cholesterol (mg), Folic acid (μg), Niacin (mg), Vitamin (RE), Vitamin B_1 (mg), Vitamin B_2 (mg), Vitamin B_6 (mg), Vitamin B_{12} (μg), Vitamin C (mg), Vitamin D (μg), Vitamin E (mg), Iron(mg), Magnesium(mg), Zinc (mg) and Selenium (μg).

Supplementary Table S1. Reference table of dietary inflammation effect

DII food parameters	Overall inflammatory effects	Global average daily intake	Standard deviation
Energy (kcal)	0.18	2056	338
Fat (g)	0.298	71.4	19.4
Dietary fiber (g)	-0.663	18.8	4.9
Carbohydrate (g)	0.097	272.2	40.0
Cholesterol (mg)	0.11	279.4	51.2
Protein (g)	0.021	79.4	13.9
Vitamin A (RE)	-0.401	983.9	518.6
Vitamin B1 (mg)	-0.098	1.7	0.66
Vitamin B2 (mg)	-0.068	1.7	0.79
Vitamin B6 (mg)	-0.365	1.47	0.74
Vitamin B12 (μg)	0.106	5.15	2.7
Vitamin C (mg)	-0.424	118.2	43.46
Vitamin D (μg)	-0.446	6.26	2.21
Vitamin E (mg)	-0.419	8.73	1.49
Folic acid (μg)	-0.19	273	70.7
Niacin (mg)	-0.246	25.9	11.77
Magnesium (mg)	-0.484	310.1	139.4
Zinc (mg)	-0.313	9.84	2.19
Iron (mg)	0.032	13.35	3.71
Selenium (μg)	-0.191	67.0	25.1

Supplementary Table S2 Characteristics of participants according to quartiles of the baseline dietary inflammatory index score.

Variable	All participants	Controls	Cases	P-values
	N=428	N =319	N =109	
Age, year	53.4 ± 16.4	53.0 ± 16.6	54.6 ± 15.9	0.39
Sex, N (%)				0.89
Male	241 (56.3)	179 (56.1)	62 (56.9)	-
Female	187 (43.7)	140 (43.9)	47 (43.1)	-
Degree of education, N (%)				< 0.001
High school and above	301(70.3)	236(74.0)	65(59.6)	301(70.3)
Senior high school	82(19.2)	49(15.4)	33(30.3)	82(19.2)
Primary education and below	45(10.5)	34(10.7)	11(10.1)	45(10.5)
Occupation, N (%)				0.16
Unemployed	83(19.4)	62(19.4)	21(19.3)	83(19.4)
Peasant	49(11.4)	19(5.9)	30(27.5)	49(11.4)
Worker	205(47.9)	166(52.0)	39(35.8)	205(47.9)
Officer	91(21.3)	61(19.1)	30(27.5)	91(21.3)
Smoking status, N (%)				< 0.01
Current smoker	71(16.6)	43(13.5)	28(25.7)	71(16.6)
Never or former smoker	357(83.4)	276(86.5)	81(74.3)	357(83.4)
Alcohol consumption, N (%)				< 0.01
Current drinker	31(7.2)	17(5.3)	14(12.8)	31(7.2)
Never or former drinker	397(92.8)	302(94.7)	95(87.2)	397(92.8)
Physical activity, N (%)				0.40
Inactive	262(61.2)	199(62.4)	63(57.8)	262(61.2)
Moderately inactive	73(17.1)	50(15.7)	23(21.1)	73(17.1)
Moderately active	78(18.2)	57(17.9)	21(19.3)	78(18.2)
Active	15(3.5)	13(4.1)	2(1.8)	15(3.5)

Note: Values are presented as mean (SD) or value (percentage) of continuous and categorical variables, respectively. P-values were obtained from t-tests or chi-square tests of independent samples, where appropriate. P-values <.0.5 was considered significant.

Supplementary Table S3. Characteristics of participants according to quartiles of the baseline dietary inflammatory index score in case-control study.

Variables	Diet Inflammation Index quartile								*P*-value
	Quartile 1		Quartile 2		Quartile 3		Quartile 4		
	-22.98~-13.12		-133.11 ~ -10.86		-10.85 ~ -8.50		-8.49 ~ 0.57		
Age, year (mean ± SD)	61.8 ± 12.1		62.9 ± 12.2		62.8 ± 11.8		63.4 ± 11.4		0.80
Sex									< 0.01
Male	48	56.5	40	43.5	58	56.3	87	69.6	-
Female	37	43.5	52	56.5	45	43.7	38	30.4	-
Degree of education									0.33
High School and above	29	34.1	30	32.6	21	20.4	31	24.8	-
Senior High school	18	21.2	17	18.5	27	26.2	27	21.6	-
Primary education and below	38	44.7	45	48.9	55	53.4	67	53.6	-
Occupation									0.24
Unemployed	7	8.2	10	10.9	16	15.6	8	6.4	-
Peasant	9	1.6	9	9.8	13	12.6	18	14.4	-
Worker	25	29.4	29	31.5	34	33.0	51	40.8	-
Officer	44	51.8	44	47.8	40	38.8	48	38.4	-
Smoking status									0.075
Current smoker	17	20.0	20	21.7	28	27.2	43	34.4	-
Never or former smoker	68	80.0	72	78.3	75	72.8	82	65.6	-
Alcohol consumption									< 0.05
Current drinker	20	23.5	18	19.6	34	33.0	49	39.2	-
Never or former drinker	65	76.5	74	80.4	69	67.0	76	62.4	-
Physical activity									0.76
Inactive	27	31.8	29	31.5	32	31.1	42	33.6	-
Moderately inactive	32	37.6	39	42.4	45	43.7	44	35.2	-
Moderately active	16	18.8	14	15.2	14	13.6	24	19.2	-
Active	10	11.8	10	10.9	12	11.7	15	12.0	-
BC patients, n (%)	14	12.4	22	19.5	35	31.0	42	37.2	< 0.001

Note: Values are presented as mean (SD) or value (percentage) of continuous and categorical variables, respectively. $P_{\text{-values}}$ were obtained from *t-tests* or *chi-square* tests of independent samples, where appropriate.

Supplementary Table S4 Characteristics of participants according to quartiles of the baseline dietary inflammatory index score in NHANES.

Variables	Diet Inflammation Index quartile								P-value
	Quartile 1		Quartile 2		Quartile 3		Quartile 4		
	-15.4 ~ -0.15		-0.16 ~ 2.29		2.30 ~ 4.49		4.50 ~ 8.64		
Age, year (mean± SD)	52.7	17.1	53.4	16.4	53.0	16.0	54.6	16.8	0.86
Sex									0.41
Male	67	62.6	56	52.3	57	53.3	61	57.0	-
Female	40	37.4	51	47.7	50	46.7	49	43.0	-
Degree of education									0.45
High School and above	79	73.8	74	69.2	68	63,6	80	74.8	-
Senior High school	19	17.8	19	17.8	28	26.2	16	15.0	-
Primary education and below	9	8.4	14	13.1	11	10.3	11	10.3	-
Occupation									0.56
Unemployed	17	15.9	24	22.4	16	15.0	26	24.3	-
Peasant	8	7.5	16	15.0	15	14.0	10	9.3	-
Worker	60	56.1	46	43.0	55	51.4	44	41,1	-
Officer	22	20.5	21	19.6	21	19.6	27	25.2	-
Smoking status									0.080
Current smoker	12	11.2	20	18.7	25	23.4	14	13.1	-
Never or former smoker	95	88.8	87	81.3	82	76.6	93	86.9	-
Alcohol consumption									0.38
Current drinker	6	5.6	9	8.4	11	10.3	5	4.7	-
Never or former drinker	101	94.4	98	91.6	96	89.7	102	95.3	-
Physical activity									0.51
Inactive	74	69.2	63	58.9	63	58.9	62	57.0	-
Moderately inactive	12	11.2	24	22.4	22	20.6	15	14.0	-
Moderately active	17	15.9	17	15.9	17	15.9	27	25.2	-
Active	4	3.7	3	2.8	5	4.7	3	2.8	-
BC patients, n (%)	19	17.4	30	27.5	28	25.7	32	29.4	0.18

Note: Values are presented as mean (SD) or value (percentage) of continuous and categorical variables, respectively. $P_{\text{-values}}$ were obtained from *t*-tests or *chi-square* tests of independent samples, where appropriate.

Supplementary Table S5. Dietary inflammatory index intake for all participants in case-control study

Variables	All participants (N = 405)	Controls (N = 292)	Cases (N = 113)	*P*
Dietary Inflammatory Index	-10.84 ± 3.48 (-22.98 ~ 0.57)	-10.84 ± 3.48 (-22.98 ~ 0.57)	-8.55 ± 3.92 (-15.82 ~ 4.74)	< 0.001

Note: Data are presented as mean ± SD (minimum ~ maximum). P_{value} represents the comparison between two groups at baseline. P <0.05, value statistically significant.

Supplementary Table S6. Dietary inflammatory index intake for all participants in NHANES study

Variables	All participants (N = 428)	Controls (N = 319)	Cases (N = 109)	*P*
Dietary Inflammatory Index	1.67 ± 3.91 (-15.36 ~ 8.64)	1.40 ± 4.10 (--15.36 ~ 8.30)	2.48 ± 3.20 (-8.49 ~ 8.64)	< 0.05

Note: Data are presented as mean ± SD (minimum ~ maximum). P_{value} represents the comparison between two groups at baseline. P <0.05, value statistically significant.
